# Effect of conjugation with organic molecules on the surface plasmon resonance of gold nanoparticles and application in optical biosensing

**DOI:** 10.1039/d1ra01842f

**Published:** 2021-07-02

**Authors:** Ehsan Koushki

**Affiliations:** Department of Physics, Hakim Sabzevari University Sabzevar 96179-76487 Iran ehsan.koushki@hsu.ac.ir ehsan.koushki@yahoo.com

## Abstract

The problem of functionalizing and coating nanoparticles with surfactants dispersed in a colloid is a prevalent case in nanoscience and related studies. It is known that surfactants could easily cause a shift in the absorption peak in metallic nanoparticles (NPs). Here, a precise theoretical model is presented to simulate the ultraviolet-visible (UV-vis) absorption spectrum of a colloid containing gold nanoparticles (Au NPs) in the presence of different surfactants. Based on the Lorentz–Drude model, this model is able to justify the fact that surfactants with a higher refractive index lead to movement of the absorption peak toward longer wavelengths (red shift). Also, relative concentrations of agents in a solvent can be analyzed using this model. The presented descriptive model illustrates gold-based biosensors with a physical point of view that leads to an increase in their efficiency. Several experimental cases are considered and are examined to calculate and compare the refractive index of the surfactants. In accordance with the results, it is found that this model is compatible with a wide range of molecular sizes, and here, the model is applied for a typical size range of micromolecules such as citrate ions to macromolecules such as polyethylene glycol (PEG) as a polyether. The suggested method revealed that it is appropriate for different surfactants with various chemical structures and refractive indexes. Utilization of approximations in this theoretical model is limited, thus, a method with the least deviation from real measurements has been introduced. The applicability of this model can be extended to practical purposes, including optical bio-sensors and detectors of organic and biological moieties such as viruses and antibodies.

## Abstract 2

The problem of functionalizing and coating nanoparticles with surfactants dispersed in a colloid is a prevalent case in nanoscience and related studies.

## Graphical abstract

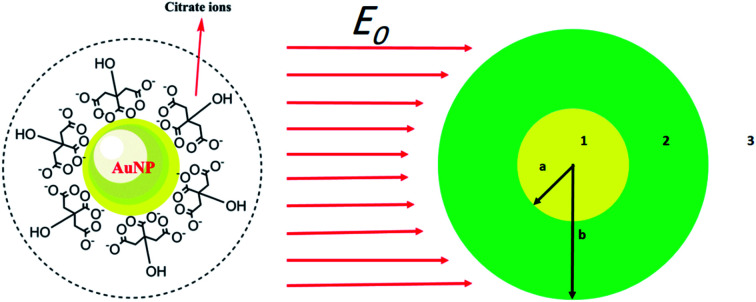

## 1. Introduction

Theoretical description of optical properties of the spherical and semi-spherical micro- and nanoparticles (MPs and NPs, respectively) have been the subject of many types of research, according to their importance for accuracy of devices, experimental procedures, and interdisciplinary studies.^1–3^ Physicists have offered different theories to describe the optical properties of the spherical solid particles dispersed in a fluid. Scattering of the electromagnetic waves from MPs has been studied using Mie theory, which can be considered a classical theory based on Maxwell's electromagnetism theory.^4–6^ This model is just applicable for the MPs in that the diameter is in the order of the incident wavelength magnitude, such as optical tweezers.^7,8^ Also, similar approximations have been used for light scattering from spherical particles introduced by Bromwich.^9^

On the other hand, conductive particles such as noble metallic particles have attracted much consideration due to their high density of free electrons and the surface plasmon resonance (SPR).^10,11^ The Lorentz–Drude model is accepted as a relatively good classical model for describing SPR in noble metallic particles and gives the complex value of electrical permittivity.^12,13^ Besides, the quantum mechanics definition of free electrons confined in a spherical potential well is helpful to understand the optical properties and resonance frequencies of conductive NPs.^14^

The models, as mentioned earlier, are focused on the optical properties of individual particles, while the collective behavior of an ensemble of particles has not been considered yet. Numerous literatures have been published to investigate the dependency of the optical permittivity of the individual particles with the optical coefficients of the host material.^15–17^ In these works, relations between the microscopic properties of the individual particles and the macroscopic optical properties of the colloid were derived. The most common models in this field are Maxwell Garnett and Bruggeman formalism.^15–17^ Reports show that the theories based on microscopic permittivity are more compatible with nanocolloids.^18,19^ In 2014, we presented a model to describe the optical properties of homogeneous nanocomposites based on Maxwell's equations.^20^ Subsequently, in 2017, this method was used in a numerical procedure to simulate the optical spectrum and dispersion curves of gold and silver nanocolloids.^21^ Theoretical results showed that the SPR peak related to gold NPs in a colloid is ultimately at 518 nm, but, at experiments, the peak was found to be in the range of about 520–530 nm.^22,23^ In many papers, this shift is considered as the gauge for detecting an agent such as drugs,^24^ viruses,^25^ and DNA.^26^

It is well-known that the peak of SPR of spherical particles can be adjusted by size and refractive index (which depends on polarizability) of the medium, which surrounds the particles. The size effect applies a boundary condition, which reduces the mean free path of the electrons and is studied in ref. 21, 27 and 28. The polarizability of the surrounding medium contains both the host medium and the surrounding agents that lead to forming a dielectric shell around the particle. The “surface-active agents” including biological ligands, alkyl chains, polymers, and other surface-active molecules, are named surfactants.^29^ Recent studies show that the effect of surfactants on the optical properties of NPs is not less than NPS themselves.^29,30^ Moreover, NPs decorated with surfactants have been noticed due to their applicability for diversified practical purposes such as medical, cleaning, purification, energy, and catalysis.^31,32^ Also, surfactants are the main factor in dispersing the particles and making a stable colloid.^33^

In previous works, some mechanisms have been introduced to specify how surfactants affect the SPR peak of noble metal NPs. On one hand, the electric field of the incident light displace the electrons from the equilibrium state and create a restoring force that leads to oscillatory motion of the electrons with a specific frequency. On the other hand, these oscillating charge carriers induce a temporary opposite polarization in the surrounding medium, which reduces the restoring force and consequently the frequency of SPR.^34^ It means a red shift in the absorption peak toward longer wavelengths (red shift) can occur. Recently, we utilized Au NPs to measure the concentration of glucose in human saliva and found that the best results can be obtained by using particles with the size of 10–13 nm.^35^

In this study, a theoretical formulation is suggested to interpret the red shift in the UV-visible spectrum. In addition, a detailed study with a physical viewpoint is presented, and results are found to be dependent on the thickness and the permittivity of the surfactants around the particles. The surfactant effects on the SPR peak are analytically described, and a simple and applicable method to find the electrical permittivity of the surfactant around the particle is offered. In other words, by using this theoretical technique, one can find the optical properties of the surfactant around the particles, which is significantly important in the characterization of the type of agents bounded to the NPs. The results were well-matched for a wide range of molecular sizes and were applied here, from micromolecules such as citrate ions to macromolecules such as polyethylene glycol (PEG). No limitation was observed due to the molecular size changes according to this theory, but the results with excess accuracy were achieved when the size of the components got bigger, components such as carbohydrate polymers. Also, the microscopic viewpoint was employed for describing electrical permittivity which its validity is widely accepted.

## 2. Experimental methods

### 2.1. Theory

In this part, a theoretical method is utilized to interpret the effect of surfactants on the SPR peak of metal NPs. The procedure is based on determining the effective permittivity of the particle coated with the surfactant and the relationship between microscopic properties and the macroscopic optical coefficients of the nanocolloid. It has been proved that Mie theory is not compatible with particles below 100 nm. The diameter of such particles is less than *λ*/4 (*λ* is the wavelength of the incident light) for visible wavelengths, where the classical scattering theories lost their validity. Mie theory has been usually used for sub-micron and micron particles.

**Fig. 1:**
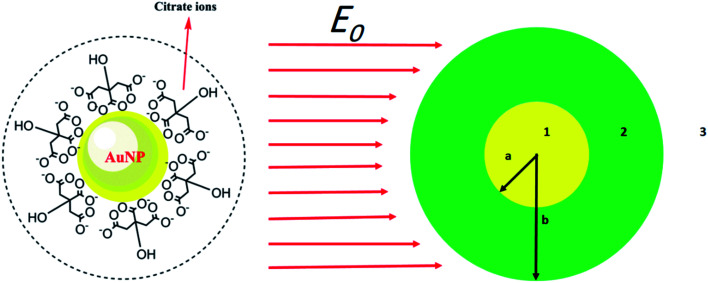
(Left) a gold nanoparticle embedded in citrate ions. (Right) schematic of a metal nanoparticle embedded in surfactant in an external electric field. The radius of the particle is *a* and for the embedded particle is *b*.

Here, we use an assumption to find the effective permittivity of a nanoparticle embedded in a dielectric shell. As we look for the UV-vis spectrum of the system, the wavelength of the applied electromagnetic field is so longer than particle size, and we can study the system through quasi-static approximation.^36^ Consider a spherical particle wrapped by a dielectric shell as shown in Fig. 1 (right), and for a typical example, we consider a corona-like shell made of citrate ions (as usually happens during reduction method) as shown in Fig. 1 (left); it can be replaced by any other organic or biological molecules. An electric field *E*_0_*ẑ* is applied to the system in the host matrix.

Solving the Laplace equation for the electric potential leads to the following potential in the host medium (medium 3):1
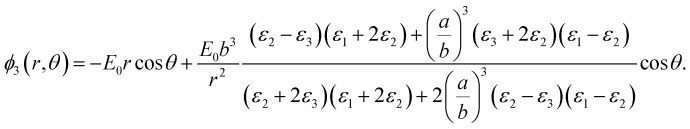


As the particle gets a net charge, an inverse square term is added to the right-hand side, which doesn't play a role in continues. Comparing eqn (1) to the electric potential of a dipole with the moment electric dipole *p*:
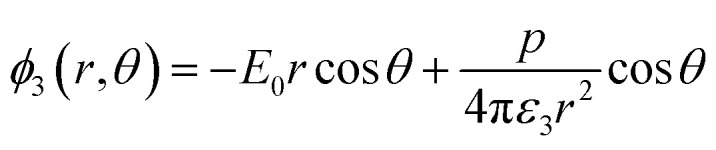
leads to the moment dipole of the system:2
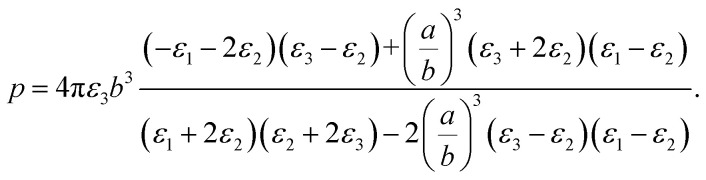


It should be noted that where the particle size is shorter enough than the wavelength, quadrupole and higher orders cannot be created.^34^ Now we consider the surfactant and the decorated particle are unique particles with the effective permittivity of *ε*_12_.3
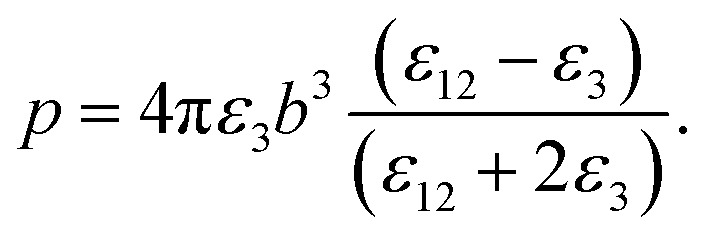


One could write the effective permittivity of the coated particle as:4
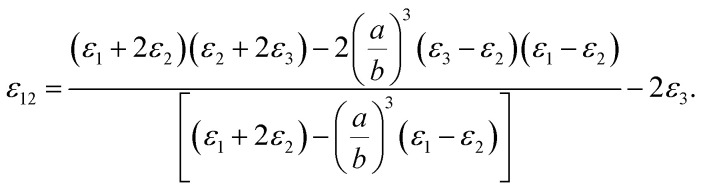


Using the following definitions;5
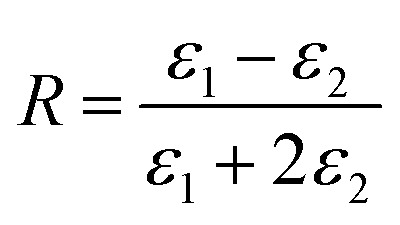
6
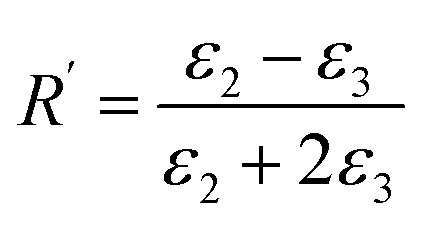
the effective permittivity of the core–shell structure is reduced to7
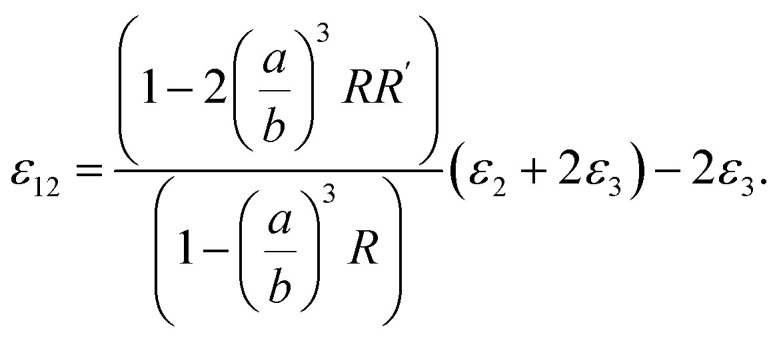
And by some algebraic simplifications, it turns to8
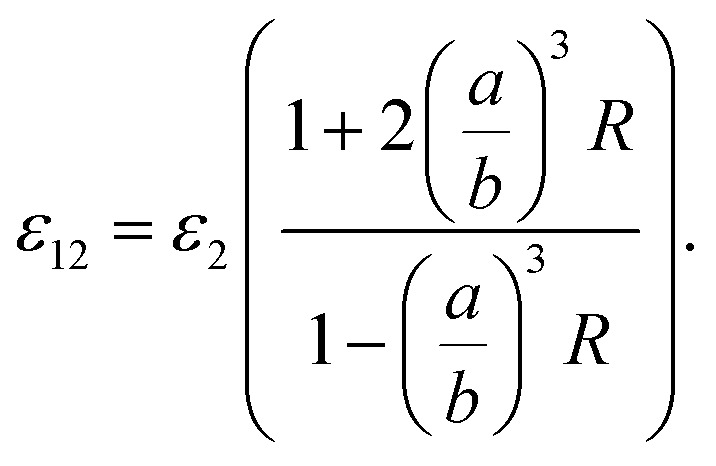


Now, we consider the core particle is not an ideal dielectric particle, and subsequently, its permittivity is a complex number and obeys the Lorentz–Drude (LD) model:^21^9
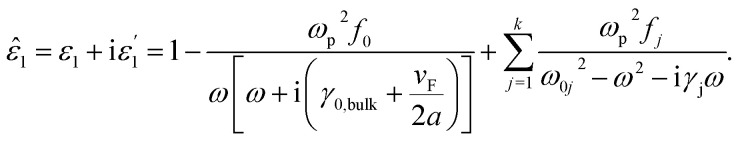
where 
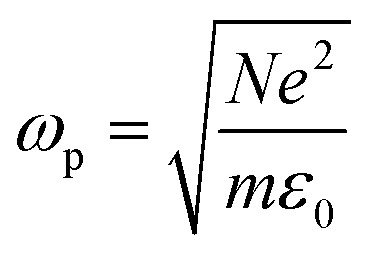
 is the plasma frequency, and *N* is the density of electrons. The second and third terms refer to free electron effects and bound electron effects, respectively. Also, *ω* and *γ*_0_ are the angular frequency of the incident wave and damping constant of free electrons, respectively. In addition, *γ*_*j*_, *f*_*j*_ = *N*_*j*_/*N* and *ω*_0*i*_ are damping constant, strength and natural frequency of oscillator associated with interband transitions of kind *j*, respectively. The quantity 
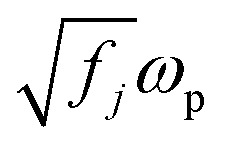
 is considered as plasma frequency associated with transitions of kind *j*.^37^ For gold, these parameters are given in Table 1. The oscillator strengths satisfy sum rule:^38^10
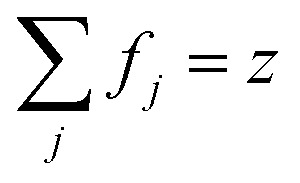
Here, *z* is the number of free and band electrons of an atom that contribute to optical responses. Furthermore, *k* is the number of possible oscillator types. More details about the damping constant have been calculated in the literature.^21^

**Table 1:** Values of LD model parameters^36^

Parameter	Au	Parameter	Au
*v*_F_ × 10^6^ (m s^−1^)^21,28^	1.4	*f*_3_	0.071
*ω*_P_ (eV)	9.03	*γ*_3_ (eV)	0.870
*f*_0_	0.760	*ω*_3_ (eV)	2.969
*γ*_0_ (eV)	0.053	*f*_4_	0.601
*f*_1_	0.024	*γ*_4_ (eV)	2.494
*γ*_1_ (eV)	0.241	*ω*_4_ (eV)	4.304
*ω*_1_ (eV)	0.415	*f*_5_	4.384
*f*_2_	0.010	*γ*_5_ (eV)	2.214
*γ*_2_ (eV)	0.345	*ω*_5_ (eV)	13.32
*ω*_2_ (eV)	0.830		

It also makes some presented parameters complex:11
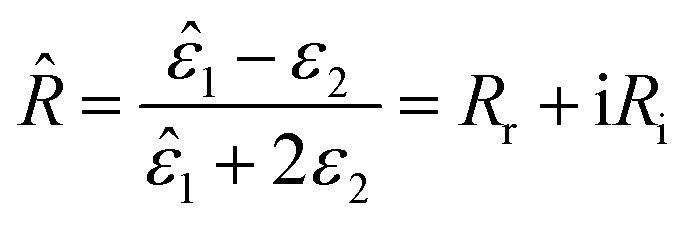
where12
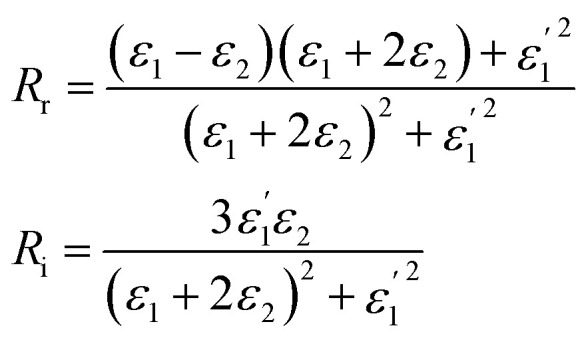
and also13
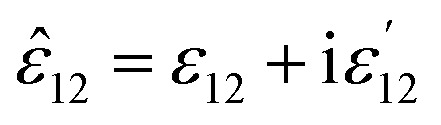
where14
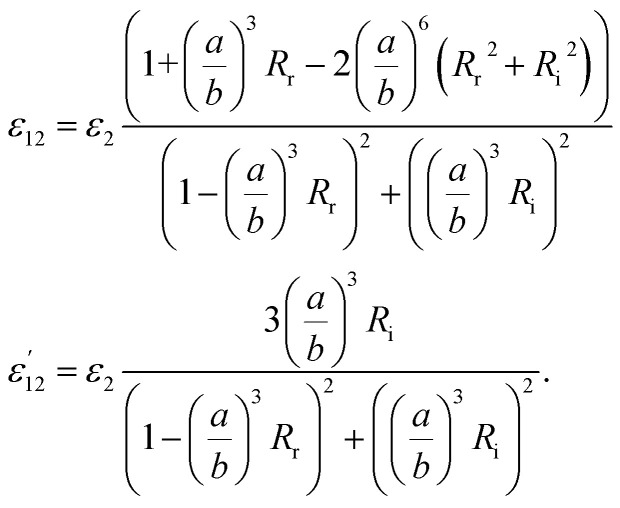


Finally, the dielectric constant and optical dispersion relations of the nanocolloid could be obtained in terms of the individual NPs properties. Real and imaginary parts of effective dielectric constant of the colloid (composite) *ε̂*_colloid_ are given by:^21^15
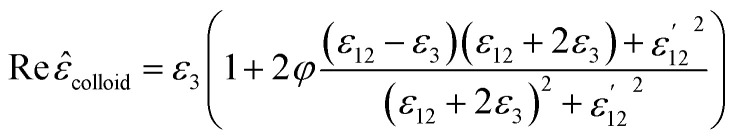
16
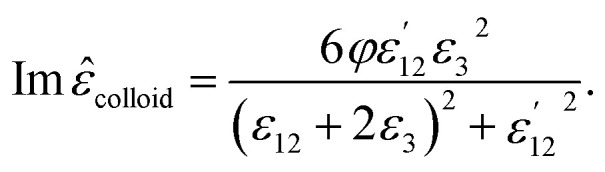
Here, *φ* is the volume fraction of the coated particles. As the denominator of the fractions is minimized, surface plasmon resonance (SPR) occurs.^39^ The refractive index (*n*) and the extinction coefficient (*κ*) of the colloid are given by:^21^17
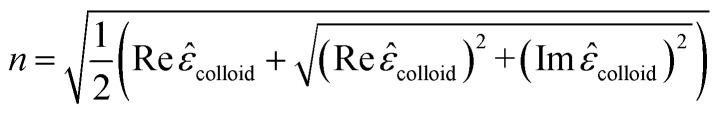
18
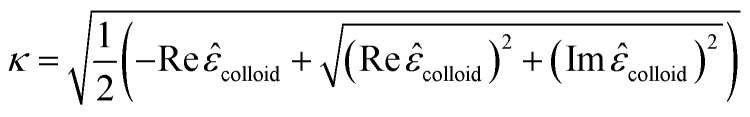
and the absorption coefficient is:19
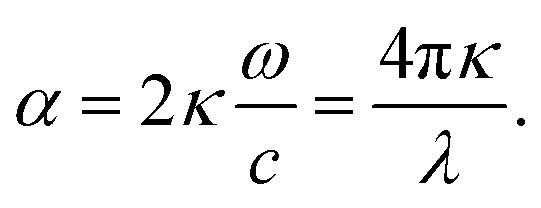


These equations give all of the optical spectral behavior of a particle coated by a dielectric surfactant. This model can be useful to find the permittivity and optical properties of the surrounding agents and surfactants embedding a conductive or semiconductor nanoparticle using a simple numerical curve fitting.

### 2.2. Synthesis of Au nanoparticles and their various characterisations

Here, this theory is tested by a simple experiment, and results are compared with similar reported ones in previous works. In this part, gold NPs dispersed in water are considered as the evidence of this theory. The synthesis method is the well-known Turkevich method.^40^ In order to synthesize Au NPs, 0.2 g of sodium citrate was poured in 20 mL distilled water and stirred for 20 min at 50 °C. After that, 6.77 mg of HAuCl_4_ was dissolved in 20 mL distilled water and was placed on a heating plate for 20 min at 100 °C till its volume reached at 15 mL. After that, 2 mL of the sodium citrate solution (which contains 0.02 g sodium citrate) was added drop-wise into the HAuCl_4_ solution for 4 seconds and placed on a heating mantel for 8 min at 100 °C.^35^

**Fig. 2:**
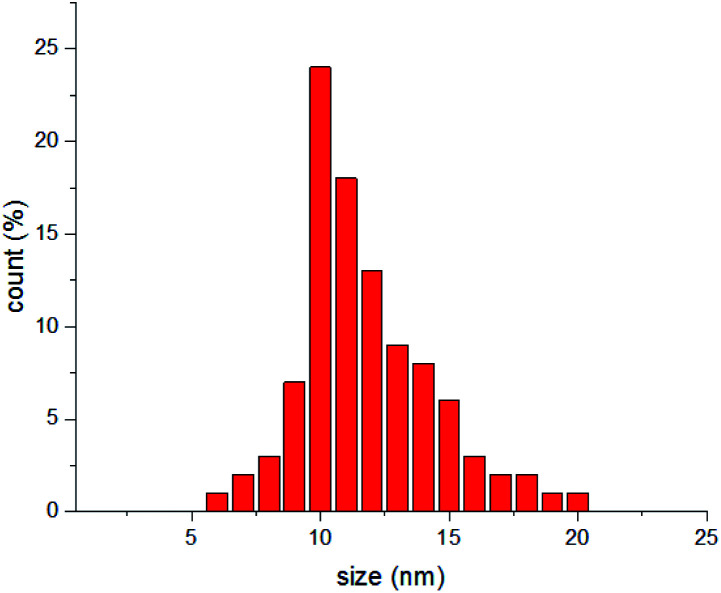
DLS measurement of synthesized Au NPs.

Dynamic light scattering (DLS) analysis has been carried out by a Malvern Zetasizer 3000, and indicated formation of particles with the mean diameter of 11.7 nm (Fig. 2).

**Fig. 3:**
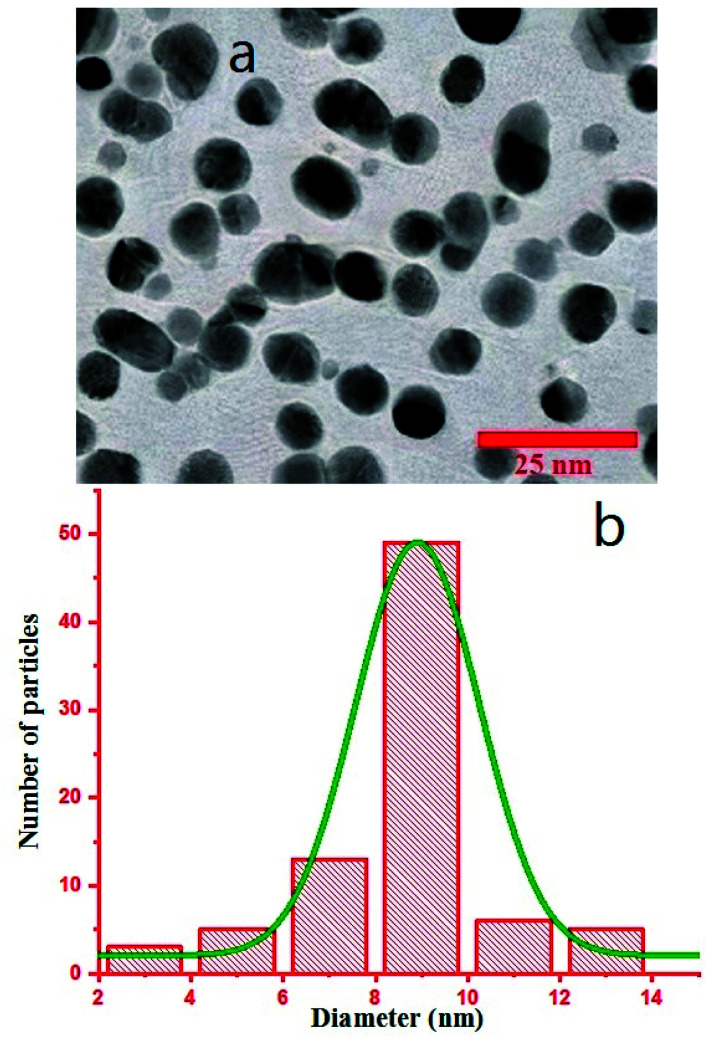
(a) TEM image of Au NPs. (b) Size distribution of the particles.

Transmission electron microscopy (TEM) was performed on a Philips CM120 with the magnification of 200 K. To monitor the morphology of Au NPs by TEM, the colloid was dispersed in ethanol using ultrasonic for 15 minutes, and a drop of the dispersed Au NPs in ethanol was poured on a carbon-coated copper grid and dried under an IR lamp, followed by inspection in the TEM device. TEM image is shown in Fig. 3a, and the size distribution of the particles was calculated *via* ImageJ software that shows the formation of particles with the mean Feret diameter of 8 nm NPs (Fig. 3b).^35^ The difference between DLS and TEM size distributions comes from the fact that DLS measures the hydrodynamic diameter of the particles that contain the surrounding agents and surfactants, but TEM gives only the Feret diameter of the particles.^41–43^ The colloid exhibited great stability, and no aggregation was observed even after several weeks. As we know, the created corona is composed of citrate ions.^40^

## 3. Results and discussion

**Fig. 4:**
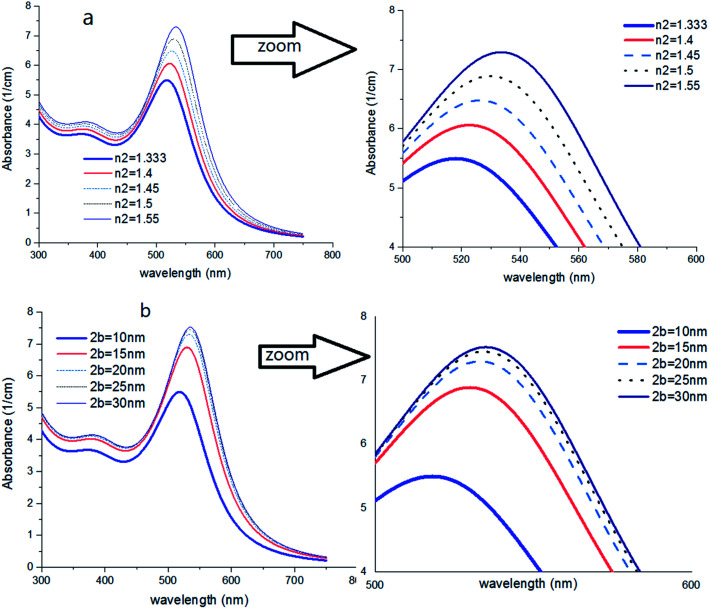
(a) Theoretical curves of UV-visible spectra of gold nanocolloids with 2*a* = 10 nm, *n*_2_ = 1.5, *n*_3_ = 1.33 (water) and different average size of hydrodynamic diameter 2*b*. (b) Theoretical curves of UV-visible spectra of gold nanocolloids with 2*a* = 10 nm, 2*b* = 15 nm, *n*_3_ = 1.33 (water) and different values of *n*_2_.

**Fig. 5:**
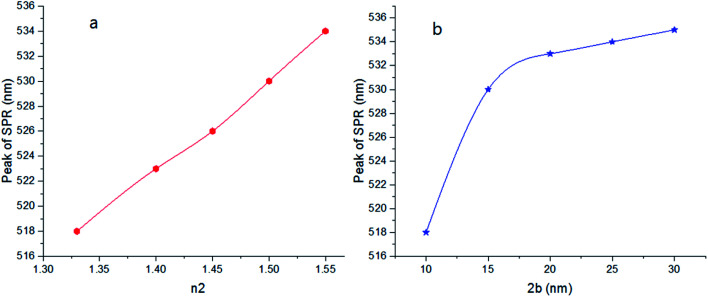
SPR peak position *versus* refractive index (a) and corona diameter (b).

**Fig. 6:**
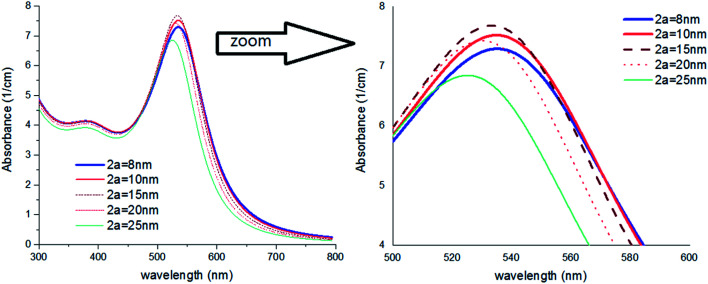
Theoretical curves of UV-visible spectra of gold nanocolloids with 2*b* = 30 nm, *n*_2_ = 1.5, *n*_3_ = 1.33 (water) and different values of 2*a*.

**Fig. 7:**
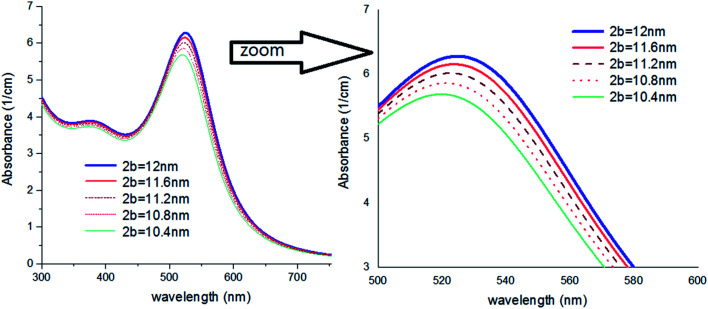
Theoretical curves of UV-visible spectra of gold nanocolloids with 2*a* = 10 nm, *n*_2_ = 1.5, *n*_3_ = 1.33 (water) and different values of 2*b*.

**Fig. 8:**
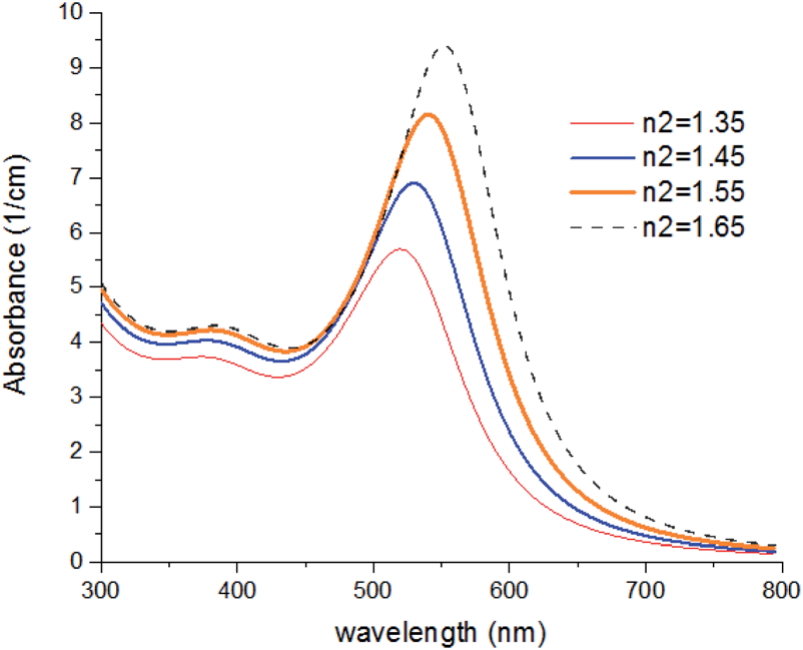
Theoretical curves of UV-visible spectra of gold nanocolloids with 2*a* = 10 nm, 2*b* = 30 nm, *n*_3_ = 1.33 (water) and different values of *n*_2_.

**Fig. 9:**
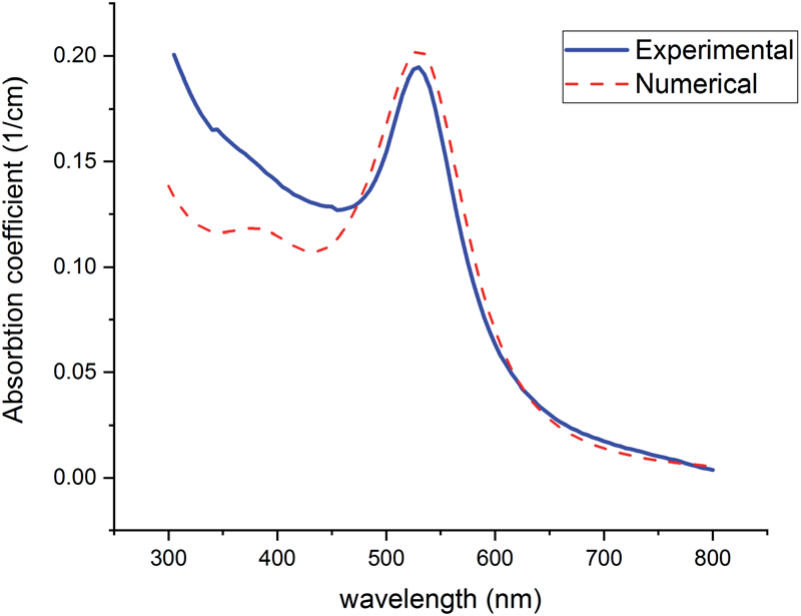
Experimental and theoretical curves of UV-visible absorption of gold NPs (with *n*_2_ = 1.5, *n*_2_ = 1.33).

First, using LD model and the parameters detailed in Table 1,^21,28,36^ the electrical permittivity of the individual particles was obtained in all wavelengths. Real and imaginary parts of the effective permittivity *ε*_12_ of the coated particles were achieved by calculating the parameters *R*_r_ and *R*_i_from eqn (12) and replacing them in eqn (14). Eqn (15)–(19) give all macroscopic properties of the nanocolloid, which is plotted in Fig. 4–9. To use the mentioned equations in plotting an absorption curve, a simple Fortran 90 programming code was written.

Fig. 4a shows the effect of the optical property of the surfactant on the SPR peak of gold nanocolloid in the UV-vis spectrum. Increasing the refractive index of the surrounding surfactant 
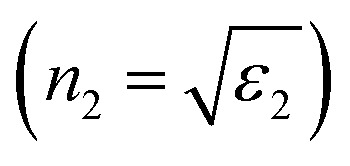
 shifts the peak to higher values. Also, increasing the diameter of the shell with a higher refractive index than the solvent can shift the peak to longer wavelengths, as depicted in Fig. 4b. In Fig. 4, the lowest curves in both the figures are the case that there is no surfactant.

Here, we can extract an important result. By investigating the trend of dependency of the SPR peak on the size of the particle, it has been reached a saturated value. It means that the sensitivity of the SPR peak to the size of the particle is high enough for thin layers of surfactants that contain microstructures. Therefore, the SPR peak position depends on both the radius and type of the agents. But for the macromolecules that form a thick surfactant layer around the particle, the changes of the SPR peak rather depend on the refractive index of the agents. This method is more straightforward for sensing macromolecules. As another important result, the width and broadening of the peak of absorption depend highly on the refractive index and thickness of agents and have a direct relationship with them. Fig. 5 shows SPR peak position *versus* refractive index and corona diameter.

As shown in Fig. 6, by increasing 2*a*, an irregular decrease appears at the red-shift of the peak. It seems that the ratio of *a*/*b* and also the refractive index *n*_2_ are the main factors to efficient red-shift.

In Fig. 7, a colloid of Au NPs was considered that are coated with different thicknesses of thin coating layers. As shown, by a very small increase of *a*/*b*, meaningful red-shift can be detected. It might be very important for scientists who wish to study the impact of much smaller additives *e.g.*, anions on the optical properties of Au NPs.^44^

In Fig. 8, results were repeated for bigger nanoparticles (2*b* = 30 nm) which confirms the previous results. The refractive index of the agents was changed from 1.35 to 1.65 which is the common range of the refractive index of liquids and solvents. Based on our knowledge, the refractive index of transparent materials is below 1.7 for liquids, 2 for solids, and refractive indexes near 2 are very rare so that the highest refractive index belongs to diamond which is 2.4.

The color of the synthesized colloid (part 2-2) was ruby, and the UV-visible spectrum is offered in Fig. 9 (Experimental). Spectrum was recorded on a UV-vis Array Spectrophotometer (PhotonixAr 2015) in the range of 300–800 nm. The SPR peak at 529 nm indicates the formation of Au NPs.

Fig. 9 shows the theoretical prediction of the SPR peak position based on the presented numerical method (red-dashed line) and experimental results (blue-solid line). Both peaks emerged at 529 nm, which confirms the great accuracy and considerable validity of this method with the least deviation from real measurements. To fit experimental data with numerical curves, using the mentioned Fortran 90 code, the refractive index was changed so much until the best fit with the experimental graphs and especially the best equation at the peak of SPR was obtained. The solvent is considered water (*n*_2_ = 1.33) in the code. In the fitted curve, volume fraction and *n*_2_ were selected 2.94 × 10^−7^ and 1.5, respectively. The value of volume fraction adjusts the height of the curves.

Below 470 nm, the experimental curve exceeds the numerical one. It can be attributed to the scattering of shorter wavelengths by the particles in this region. However, this phenomenon occurs below 470 nm where is far from the SPR (520–530 nm), which is focused on in this study.

This model was applied for some other samples whose details have been reported in previous articles.^24,45–48^ In Table 2, the refractive index of the coating shell (*n*_2_) is obtained for the samples. The near values of the refractive index of citrate ions (in cases 1, 2, 6, and 8) can be assigned to considerable validity of this method.

**Table 2:** The calculated refractive index of some usual surfactants with this model. The reported values of the Feret and hydrodynamic diameters are the mean values

	Capping agents	Feret diameters diameter of the particle (TEM) 2*a* (nm)	Hydrodynamics diameter (DLS) 2*b* (nm)	Peak of SPR (UV-visible) (nm)	The calculated refractive index of coating shell (*n*_2_)	Ref.
1	Citrates ions	7.5	11.5	529	1.51	This work
2	Citrates ions	25	28	525	1.51	45
3	Poly ethylene glycol	25	42	526	1.42	45
4	VHH-122 antibodies	25	45	527	1.45	46
5	AAP (a triptan-family drug)	10	13.9	544	1.73	24
6	Citrate ions	25	29	521	1.49	47
7	Antibody	25	86.2	526	1.41	47
8	Citrate ions	21.7	24.5	522	1.50	48
9	Cetuximab (C225)	21.7	41.1	525	1.41	48

It should be noted that the calculated refractive index of the capping agent (*n*_2_) should have been assigned to both the solution and the agents which environed the particle. It should not be related to the agents only. Also, the concentration of the agents and the arrangement direction of them in the solution are very important. Orientation of the bimolecular or organic agents influences the optical response of dielectric composite.^49,50^ Around a nanoparticle, agents have an anisotropy radial orientation which is different from parallel orientation. Furthermore, conformation preferences of ligands can influence the binding strength between the particle and molecules and consequently affects the plasmon peak, especially for chain molecules such as long-chain surfactants, proteins, and carbohydrates.^29^

Here we give proof of the correctness of the mentioned calculations. As we know, the dielectric dipole moment that is a microscopic parameter has a direct relation with macroscopic parameters such as electric constant and refractive index.^51^ The dielectric dipole moment of a poly ethylene glycol changes between 2.38 D (monomer) to 3.65 D (heptamer),^52^ but this quantity is 38.48 D for citric acid^53^ and is certainly higher for its ion. It is in good agreement with our results, as shown in Table 2.

It is interesting to investigate the validity of the model for a solvent expects to water, so it will be checked for Au NPs dispersed in chloroform. S. Elhani1 *et al.* provided a colloid of Au NPs in chloroform by removing the particles from water and transferring them into chloroform as a colloidal solution.^54^ It was done by using surfactants that cover the surface of Au-NPs and separate them from water.^54,55^ In brief, the surfactants used for the transfer are amphiphilic molecules with a hydrophobic head that binds to Au NPs, and a hydrophilic tail, which like water. By adding Au nanocolloid to the desired solvent containing appropriate surfactants, the transfer process would have happened. The transfer mechanism is a very important mechanism that enables us to obtain colloids from Au NPs with a wide variety of solvents and consequently different colors.^54,55^ The reason for these color changes is understandable with this model. For example, in ref. 50, the production of the transfer process of Au NPs in chloroform solution is a colloid of Au NPs coated with the mixture of two main surfactants; (hexadecyl) trimethylammonium bromide (CTAB) and tetraoctylammonium bromide (TOAB) citrate ions, and probably a percent of citrate ions. The refractive index of all of these ionic surfactants is about 1.41,^56^ and the refractive index of chloroform is 1.445.

**Fig. 10:**
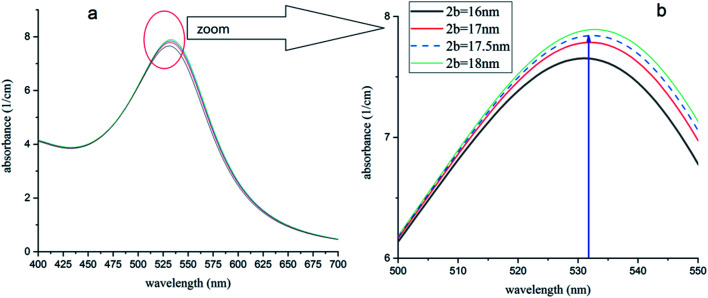
(a) Absorption spectra of Au-NPs (∼15 nm) in chloroform with different dimensions of capping agent shell. (b) The peak of 532 nm (ref. 54) is obtained for 2*b* = 17.5 nm and is in good agreement with ref. 54.

The model was used for this case. Fig. 10 shows the simulated UV-visible spectra of the Au NPs with capping agents of a mixture of CTAB, TOAB and citrate ions (*n*_2_ = 1.41), dispersed in chloroform. Different average sizes of shell dimension (2*b*) were used in simulations. As shown, for 2*b* = 17.5 nm, the peak was obtained at 532 nm, which is equal to the value obtained by the experiment^54^ and confirms a shift of plasmon resonance to 532 nm. In other words, the best fit of experimental and numerical peaks is obtained for 2*b* = 17.5 nm which is the most probable dimension of the capping agent.

Optical biosensors are analytical devices often including a source light and a detecting device which measure or monitor a biological component. They are capable of detecting biomolecules in a complex sample by converting the optical signal to an optical or electrical signal which can be further processed to the concentration of the biomolecules, such as glucose, antibodies, enzymes and hormone receptors.

Different optical mechanisms have been used for this purpose including SPR, refractive index, optical waveguide, optical resonator, optical fiber, and optical absorption.^57^ In this study, absorption coefficient of a metal nanocolloid has been used as the sensing technique which is based on changing the SPR of metal particles by refractive index of biomolecules as capping agents. This technique can be used as an optical biosensor with good performance in detecting biomolecules and promote acceptable progress in clinical diagnostics, drug process control, drug delivery and discovery, and environmental monitoring.

## 4. Conclusion

In conclusion, a theoretical method was offered in order to simulate the absorption spectra of gold nanoparticles dispersed in water. The model was focused on the microscopic permittivity of the nanoparticles and the surrounding agents and their contribution to the optical properties of the nanocolloid. For the first time, the physical reason of the SPR peak shift by capping agents was investigated. Also, by using this model and the wavelength of the absorption peak, one can obtain the refractive index of the surfactant that depends on the type and density of the capping agents. This model offers an accurate method that can be used in biosensing and surfactant science, based on SPR of Au NPs.

## Conflicts of interest

The author wish to confirm that there are no known conflicts of interest associated with this publication, and there has been no significant financial support for this work that could have influenced its outcome.

## Supplementary Material

## References

[cit1] Alvare M. M., Khoury J. T., Schaaff T. G., Shafgullin M. N., Vezmar I., Whetten R. L. (1997). Optical absorption spectra of nanocrystal gold molecules. J. Phys. Chem. B.

[cit2] El-Sayed I. H., Huang X., El-Sayed M. A. (2005). Surface plasmon resonance scattering and absorption of anti-EGFR antibody conjugated gold nanoparticles in cancer diagnostics: Applications in oral cancer. Nano Lett..

[cit3] Daniel M. C., Astruc D. (2004). Gold nanoparticles: Assembly, supramolecular chemistry, quantum-size-related properties, and applications toward biology, catalysis, and nanotechnology. Chem. Rev..

[cit4] Bohren C. and Huffman D., Absorption and Scattering of Light by Small Particles, Wiley Science Series, Wiley, 2008

[cit5] Mie G. (1908). Beiträgezur Optiktrüber Medien, speziell kolloidaler Metallösungen. Ann. Phys..

[cit6] Logan N. A. (1965). Survey of some early studies of the scattering of plane waves by a sphere. Proc. IEEE.

[cit7] Ranha Neves A. A., Cesar C. L. (2019). Analytical calculation of optical forces on spherical particles in optical tweezers: tutorial. J. Opt. Soc. Am. B.

[cit8] Salandrino A., Fardad S., Christodoulides D. N. (2012). Generalized Mie theory of optical forces. J. Opt. Soc. Am. B.

[cit9] Gouesbet G., Maheu B., Gréhan G. (1988). Light scattering from a sphere arbitrarily located in a Gaussian beam, using a Bromwich formulation. J. Opt. Soc. Am. A.

[cit10] Li R., Wang D., Guan J., Wang W., Ao X., Schartz G. C., Schaller R., Odom T. W. (2019). Plasmon nanolasing with aluminum nanoparticle arrays. J. Opt. Soc. Am. B.

[cit11] Link S., El-Sayed M. A. (1999). Size and temperature dependence of the plasmon absorption of colloidal gold nanoparticles. J. Phys. Chem. B.

[cit12] Kittle C. , Introduction of Solid State Physics, Wiley, New York, 7th edn, 1996

[cit13] Ashcroft N. W. and Mermin N. D., Solid State Physics, Saunders College Publishing, New York, 1st edn, 1976

[cit14] Hache F., Richard D., Flytzanis C. (1986). Optical properties of small metal particles: surface-mediated resonance and quantum size effects. J. Opt. Soc. Am. B.

[cit15] Wen W., Huang X., Sheng P. (2008). Electrorheological fluids: structures and mechanisms. Soft Matter.

[cit16] Bergman D. J. (1982). Rigorous bounds for the complex dielectric constant of a two-component composite. Ann. Phys..

[cit17] Sihvola A. , Mixing rules with complex dielectric coefficients, Surface sensing technologies and applications, 2000, vol. 1, pp. 393–415

[cit18] Allen P. B. (2004). Dipole interactions and electrical polarity in nanosystems: The clausius-Mossotti and related models. J. Chem. Phys..

[cit19] Vanzo D., Topham B. j., Soos Z. G. (2015). Dipole-field sums, Lorentz factors and dielectric properties of organic molecular films modeled as crystalline arrays for polarizable points. Adv. Funct. Mater..

[cit20] Koushki E., MajlesAra M. H. (2014). Modeling electrical and optical spectral responses of homogeneous nanocomposites. Phys. E.

[cit21] Koushki E., Farzaneh A. (2017). Numerical simulation of optical dispersion, group velocity, and waveguide properties of gold and silver nanocolloids and hybrids. Colloid Polym. Sci..

[cit22] Jain P. K., Seok Lee K., El-Sayed I. H., El-Sayed M. A. (2006). Calculated Absorption and Scattering Properties of Gold Nanoparticles of Different Size, Shape, and Composition: Applications in Biological Imaging and Biomedicine. J. Phys. Chem. B.

[cit23] Schaffler M., Semmler-Behnke M., Sarioglu H., Takenaka S., Wenk A., Schleh C., Hauck S. M., Johnston B. D., Kreyling W. G. (2013). Serum protein identification and quantification of the corona of 5, 15 and 80 nm gold nanoparticles. Nanotechnology.

[cit24] Rawat K. A., Surati K. R., Kailasa S. K. (2014). One-pot synthesis of gold nanoparticles by using 4-aminoantipyrine as a novel reducing and capping agent for simultaneous colorimetric sensing of four triptan-family drugs. Anal. Methods.

[cit25] Shawky S. M., Awad A. M., Allam W., Alkordi M. H., EL-Khamisy S. F. (2017). Gold aggregating gold: a novel nanoparticle biosensor approach for the direct quantification of hepatitis C virus RNA in clinical samples. Biosens. Bioelectron..

[cit26] Zhang Y., Fei W.-W., Jia N.-Q. (2012). A facile method for the detection of DNA by using gold nanoparticle probes coupled with dynamic light scattering. Nanoscale Res. Lett..

[cit27] Link S., El-sayed M. (2000). Shape and size dependence of radiative, non-radiative and photothermal properties of gold nanocrystals. Int. Rev. Phys. Chem..

[cit28] Warrier P., Teja A. (2011). Effect of particle size on the thermal conductivity of nanofluids containing metallic nanoparticles. Nanoscale Res. Lett..

[cit29] Heinz H., Pramanik Ch., Heinz O., Ding Y., Mishra R. K., Marchon D., Flatt R. J., Estrela-Lopis I., Liop J., Moya S., Ziolo R. F. (2017). Nanoparticle decoration with surfactants: molecular interactions, assembly, and applications. Surf. Sci. Rep..

[cit30] Parhizkar M., Edirisinghe m., Stride E. (2015). The effect of surfactant type and concentration on the size and stability of microbubbles produced in a capillary embedded T-junction device. RSC Adv..

[cit31] Smeets P. J. M., Cho K. R., Kempen R. G. E., Sommerdijk N., De Yoreo J. J. (2015). In situ TEM shows ion binding is key to directing CaCO_3_ nucleation in a biomimetic matrix. Nat. Mater..

[cit32] Xavier J. R., Thakur T., Desai P., Jaiswal M. K., Sears N., Cosgriff-Hernandez E., Kaunas R., Gaharwar A. K. (2015). Bioactive nanoengineered hydrogels for bone tissue engineering: a growth-factor-free approach. ACS Nano.

[cit33] Patel J., Nemcova L., Maguire P., Graham W. G., Mariotti D. (2013). Synthesis of surfactant-free electrostatically stabilized gold nanoparticles by plasma-induced liquid chemistry. Nanotechnology.

[cit34] Evanoff Jr D. D., Chumanov G. (2005). Synthesis and optical properties of silver nanoparticles and arrays. Chemphyschem.

[cit35] Koushki E., Mirzaei Mohammadabadi F., Baedi J., Ghasedi A. (2020). The effects of glucose and glucose oxidase on the UV-vis spectrum of gold nanoparticles: A study on optical biosensor for saliva glucose monitoring. Photodiagn. Photodyn. Ther..

[cit36] Sancho-Parramon J. (2009). Surface plasmon resonance broadening of metallic particles in the quasi-static approximation: a numerical study of size confinement and interparticle interaction effects. Nanotechnology.

[cit37] Rakić A. D., Djurišić A. B., Elazar J. M., Majewski M. L. (1998). Optical properties of metallic films for vertical-cavity optoelectronic devices. Appl. Opt..

[cit38] Jackson J. D. , Classical electrodynamics, Wiley, New York, 3rd edn, 1998

[cit39] Takeda Y., Plaksin O. A., Lu J., Kishimoto N. (2006). Optical nonlinearity of metal nanoparticle composites fabricated by negative ion implantation. Vacuum.

[cit40] Turkevich J., Stevenson P. L., Hillier J. (1951). A study of the nucleation and growth process in the synthesis of colloidal gold. Discuss. Faraday Soc..

[cit41] Zheng T., Cherubin P., Cilenti L., Teter K., Huo Q. (2016). A simple and fast method to study the hydrodynamic size difference of protein disulfide isomerase in oxidized and reduced form using gold nanoparticles and dynamic light scattering. Analyst.

[cit42] Hinterwirth H., Wiedmer S., Moilanen M., Lehner A., Allmaier G., Waitz T., Lindner W., Lämmerhofer M. (2013). Comparative method evaluation for size and size-distribution analysis of gold nanoparticles. J. Sep. Sci..

[cit43] Hinterwirth H., Wiedmer S., Moilanen M., Lehner A., Allmaier G., Waitz T., Lindner W., Lämmerhofer M. (2013). Comparative method evaluation for size and size-distribution analysis of gold nanoparticles. J. Sep. Sci..

[cit44] Rehbock C., Huehn D., Carrillo-Carrion C., de Aberasturi D., Merk V., Barcikowski S., Parak W. (2014). Interaction of colloidal nanoparticles with their local environment: the (ionic) nanoenvironment around nanoparticles is different from bulk and determines the physico-chemical properties of the nanoparticles. J. R. Soc., Interface.

[cit45] Brun E., Sicard – Roselli C. (2014). Could nanoparticle corona characterization help for biological consequence prediction?. Cancer Nanotechnol..

[cit46] Ashton J. R., Gottlin E. B., Patz, Jr E. F., West J. L., Badea C. T. (2018). A comparative analysis of EGFR-targeting antibodies for gold nanoparticle CT imaging of lung cancer. PLoS One.

[cit47] Pandey S. K., Suri C. R., Chaudhry M., Tiwaria R. P., Rishi P. (2012). A gold nanoparticles based immuno-bioprobe for detection of Vi capsular polysaccharide of *Salmonella entericaserovar Typhi*. Mol. BioSyst..

[cit48] Zhu C., Wang L., Cal Y., Wang G., Xu H., Wan Y., Zheng Q. (2018). Enhanced radiation effect on SMCC7721 cells through endoplasmic reticulum stress induced by C225-GNPs *in vitro* and *in vivo*. Oncol. Lett..

[cit49] Biswas P. K. (1984). The mechanical and optical properties of oriented fibres of semicrystalline polymers. Colloid Polym. Sci..

[cit50] Ward I. M. Structure and Properties of Oriented Polymers, Springer, Netherlands, 2nd edn, 1997

[cit51] Boyd R. W. , Nonlinear Optics, Academic Press, 3rd edn, 2007

[cit52] Uchida T., Kurita Y., Koizumi N. (1956). Dipole Moments and the Structures of Polyethylene Glycol. J. Polym. Sci..

[cit53] Ali H., Carpenter-Warren C. L., Slawin A. M. Z., Haddad A. (2019). Structure and Properties evolution with inorganic and organic acids of a new organo-chlorocadmate compound (C_6_H_20_N_3_)_2_[Cd_2_Cl_10_]: Theoretical approach. J. Mol. Struct..

[cit54] Elhani1 S., Ishitobi H., Inouye Y., Ono A., Hayashi S., Sekkat Z. (2020). Surface enhanced Visible Absorption of Dye Molecules in the near-field of Gold nanoparticles. Sci. Rep..

[cit55] Joshi C. P., Bigioni T. P. (2014). Model for the Phase Transfer of Nanoparticles Using Ionic Surfactants. Langmuir.

[cit56] Begum F., Yousuf M., Mollah A., Rahman M. M., Abu Bin Hasan Susan M. (2015). Acid Hydrolysis of Bromazepam Catalyzed by Micelles, Reverse Micelles, and Microemulsions. J. Chem..

[cit57] Chen C., Wang J. (2020). Optical biosensors: an exhaustive and comprehensive review. Analyst.

